# Curvilinear Effects of Extraversion on Socialization Outcomes Among Chinese College Students

**DOI:** 10.3389/fpsyg.2021.652834

**Published:** 2021-06-03

**Authors:** Yingxin Deng, Huitian Chen, Xiang Yao

**Affiliations:** ^1^Beijing Key Laboratory of Behavior and Mental Health, School of Psychological and Cognitive Sciences, Peking University, Beijing, China; ^2^Middlebury College, Middlebury, VT, United States

**Keywords:** curvilinear relationship, extraversion, social acceptance, depression, freshmen adjustment

## Abstract

The authors examine the too-much-of-a-good-thing effect (TMGT effect) in a model showing that extraversion has a curvilinear relationship with social acceptance and depression. A study of 371 freshmen in a Chinese university showed that extraversion had a curvilinear relationship with social acceptance, such that the relationship was significantly positive from lower to moderate levels of extraversion, but the positive relationship leveled off at higher levels of extraversion. Extraversion also had a curvilinear relationship with depression, such that the relationship was significantly negative from lower to moderate levels of extraversion, but the negative relationship leveled off at higher levels of extraversion. The study indicates that beyond a certain point, the beneficial effects of extraversion on socialization outcomes were diminished. That is, higher levels of extraversion were not associated with more positive socialization outcomes (though they were not associated with worse outcomes either) when extraversion exceeded a certain point. Implications of theory and practice, and limitations and directions for future research, are discussed.

## Introduction

Extraversion, defined as “a dimension of personality reflecting individual differences in the tendencies to experience and exhibit positive affect, assertive behavior, decisive thinking, and desires for social attention” (Wilt and Revelle, 2017, p. 57), has been found to be positively related to interpersonal relationships (Hogan et al., 1997; Jensen-Campbell et al., 2002; Lubbers et al., 2006) and psychological wellbeing (Costa and McCrae, 1980; Lee et al., 2008). As such, extraversion is considered an important predictor of freshmen adjustment to the college environment (Wang et al., 2013; Klimstra et al., 2018; Zhang et al., 2021).

However, whether extraversion is altogether advantageous remains tentative. Although extraversion was found to positively predict social relations (Jensen-Campbell et al., 2002; Lubbers et al., 2006), individuals exhibiting high levels of extraversion tend to be dominant, impulsive, and eager to be at the center of social attention (Depue and Collins, 1999; Ashton et al., 2002; Roberts et al., 2006; Shao et al., 2013; Hu et al., 2019), which would not always result in favorable social interactions (Eysenck and Eysenck, 1975; Zee et al., 2013). For instance, they may experience more antagonistic conflict in competing for social positions (Lund et al., 2007; Anderson and Shirako, 2008), especially if they insist on their viewpoints while neglecting the perspectives and interests of others (Grant, 2013; Hu et al., 2019). They may limit the time and energy they devote to close relationships (Ashton and Lee, 2007; Gurven et al., 2014) because they prefer to expand their social network to gain increased social attention (Corr and Matthews, 2009). As such, people possessing high levels of extraversion may not be socially accepted well and are more likely to get into burnout and exhaustion (Eastburg et al., 1994). Based on the above justifications, extraversion would have complex effects on socialization outcomes beyond indications revealed from zero-order correlations or simple linear regression models.

The “too-much-of-a-good-thing” effect (TMGT effect) was defined as “when ordinarily beneficial antecedents…reach inflection points after which their relations with desired outcomes…cease to be linear and positive” (Pierce and Aguinis, 2013, p. 315). For instance, conscientiousness has a positive impact on job performance over some range, but once a certain threshold is crossed, increased levels of conscientiousness have an increasingly negative impact on job performance (Le et al., 2011). Other studies also show that some personality traits, such as conscientiousness and neuroticism, are curvilinearly related to task-relevant performance (Le et al., 2011; Carter et al., 2014; Uppal, 2017; Yuan et al., 2018). Given that the interpersonal relationship and psychological wellbeing are two other key components of newcomer socialization beyond task performance (Chickering, 1969; Mattanah et al., 2010; Klimstra et al., 2018; Deng and Yao, 2020), it is important to understand the effects of personality trait on social and emotional adjustment for freshmen. However, to the best of our knowledge, no research has been concerned about whether desirable traits, such as extraversion, have curvilinear effects on the interpersonal relationship and psychological wellbeing.

Social acceptance indicates the extent of social adjustment to a new environment (Bauer et al., 2007). When individuals feel socially accepted, they form “relatively stable cognitive appraisals that others care for and value” that their peer group accepts their attitudes and behaviors (Brock et al., 1998, p. 1). Depression is considered an important socialization indicator of the psychological wellbeing of students (Hintz et al., 2015; Duan and Bu, 2019), which mainly reflects the maladaptive emotional states of college freshmen in their new environment. The TMGT effect posits that overly extraverted freshmen are likely to utilize their social dominance and skills to approach their personal goals, which may not be interpersonally or team orientated (Grant et al., 2011), resulting in lower levels of social acceptance. In addition, extremely extraverted individuals are more likely to be burnt out or exhausted due to the frequency of social interactions and the pursuit of social attention (Eastburg et al., 1994). Failing to receive the desired social attention, they may easily fall into depressive emotion (Corr and Matthews, 2009). In contrast, moderately extraverted freshmen would be regarded as competent and likable, which indicates they are more likely to be socially accepted and have more resistance to depression.

Our study makes three contributions to the literature. First, the curvilinear effect of extraversion has long been neglected by socialization researchers. The current study fills the gap in the literature by examining the curvilinear effects of the important personality factor in the Big Five personality traits framework, extraversion, on two important socialization outcomes: social acceptance and depression. Second, previous studies have paid much attention to the curvilinear effect of personality traits on task-relevant outcomes. However, we shed the spotlight on two other important socialization outcomes, interpersonal relationship (i.e., social acceptance) and psychological wellbeing (i.e., depression), and argue that extraversion has the curvilinear effect on a broader set of socialization outcomes. Third, our study provides empirical evidence to the “too much of a good thing” of personality factor on newcomer adjustment by showing the curvilinear effect of extraversion on the socialization process.

### Extraversion and Social Acceptance: A Curvilinear Hypothesis

Extraverted people are generally assertive, excitement-seeking, talkative, and people-oriented (Costa and McCrae, 1992). They are motivated to engage in social interactions, such as making friends at school (Okun and Finch, 1998; Zhang et al., 2021), helping others, and managing the impressions they convey (Chiaburu et al., 2015). Such characteristics could lead to greater social acceptance and adaptation. Moreover, the extraverts are considered to have good interpersonal skills because they are enthusiastic and warm (Curşeu et al., 2019; Hu et al., 2019). For instance, they are more likely to exhibit positive attitudes and share credits in teamwork (Hogan and Holland, 2003; Curşeu et al., 2019). As extraverted freshmen are inclined to be “affectionate and friendly” (Hu et al., 2019, p. 1371), they are more likely to make friends, achieve acceptance, and maintain good social relationships with peers in the new environment (Curşeu et al., 2019; Hu et al., 2019).

The extraversion literature, however, has neglected the possibility that extremely extraverted individuals could be annoying, overly dominant, and too impulsive in their desires while seeking attention (Shao et al., 2013; Zee et al., 2013). Extraversion has been found to be positively associated with relationship conflict (Bono et al., 2002). Overly extraverted individuals tend to be assertive and interpersonally dominant (Volk et al., 2021), so they are more likely to exert control over their surroundings (Grant et al., 2011) and to be more concerned about the extent to which they could attract the social attention of others (Ashton et al., 2002). Assertive and dominant individuals who lack formal designated power are even more likely to draw negative peer reactions (Hu et al., 2019). When extremely extraverted individuals interact in groups, they are perceived to be less reliable because they are sometimes uncooperative, overly impulsive, and make conclusions too early (Quilty et al., 2014). In this case, higher levels of extraversion may not be helpful in promoting social acceptance. As such, the relationship between extraversion and social acceptance is likely to be nonlinear. To sum up, we hypothesize:

*Hypothesis* 1: Extraversion has an inverted U-shaped relationship with social acceptance, such that the relationship is initially positive but becomes less positive as extraversion increases.

### Extraversion and Depression: A Curvilinear Hypothesis

Extraverted individuals are often described as outgoing, cheerful, and sociable (Eysenck and Eysenck, 1975) who have more confidence in the future because of their strong social support (Baryshnikov et al., 2018). As such, when they face difficulties, they would stay optimistic and adopt positive coping strategies (Sulea et al., 2015; Nagata et al., 2019). Moreover, extraversion has been found to be positively related to positive affect (Lucas and Fujita, 2000; Fleeson et al., 2002), with which people would experience less stress and have more positive feelings (Naragon-Gainey et al., 2009; Backmann et al., 2019). As extraverted individuals tend to be more resilient (Campbell-Sills et al., 2006; Gong et al., 2020), they are more likely to successfully overcome the adversities and maintain psychological wellbeing, resulting in lower levels of depression (Backmann et al., 2019; Gong et al., 2020).

However, researchers have not yet studied whether extraversion beyond certain levels may cause highly extraverted students to feel burnt out and depressed. Extraverted students may easily make peers view them as disrespectful and aggressive (Chen et al., 1995). If the increasing needs for social support are unfulfilled (Corr and Matthews, 2009), extraverted students may have more depressive emotions. Furthermore, being extraverted costs a lot of energy since they are more active and talkative in their daily lives (Hogan et al., 1997). Thus, it is more easy for overly extraverted individuals to fall into burnout and exhaustion (Eastburg et al., 1994). In this case, higher levels of extraversion may not be helpful in reducing depression. As such, the relationship between extraversion and depression is likely to be nonlinear. To sum up, we hypothesize:

*Hypothesis* 2: Extraversion has a U-shaped relationship with depression, such that the relationship is initially negative but becomes less negative as extraversion increases.

## Methods

### Participants and Procedure

We recruited 393 freshmen from a comprehensive university in China through campus internet postings or emails. Before we administered the survey, we emphasized that participation was voluntary and confidential. Those who completed all survey rounds were paid 30 RMB. The first month after participants entered the university, they completed a survey measuring personality traits. Three months later, they completed the social acceptance and depression scales. 371 freshmen completed all questionnaires, a response rate of 94.40%. Mean age of participants was 18.10 (standard deviation (SD) = 0.69); 50.5% were men; six failed to specify gender.

### Measures

We used the back-translation approach (Brislin, 1980) to ensure that the English-language measures were accurately translated into Chinese. Items were slightly altered to reflect an academic context.

Extraversion was measured using the Mini-Markers developed by Saucier (1994). Mini-Markers is a shortened version of Goldberg’s unipolar Big-Five Markers (Goldberg, 1992). The Extraversion scale contains eight adjective markers [four positive adjectives such as “Talkative” and four negative adjectives (reversed coded) such as “Bashful”]. Respondents rated each marker using a Likert scale ranging from 1 (*extremely inaccurate*) to 7 (*extremely accurate*). The Cronbach’s alpha coefficient for extraversion was 0.84.

Social acceptance was measured using the 12-item scale (1 = *strongly disagree* to 7 = *strongly agree*) developed by Brock et al. (1998). A sample item: “I am very important in the lives of my classmates.” The Cronbach’s alpha coefficient for social acceptance was 0.86.

Depression was measured using Van de Velde et al. (2010) eight-item CES-D eight scale (1 = *never* to 4 = *almost every moment*). A sample item: “I could not get going.” The Cronbach’s alpha coefficient for depression was 0.86.

Control variables were measured in the first month after freshmen entered the university. Big Five personalities have been found to be linked to socialization outcomes (e.g., Wang et al., 2013), so we controlled for all Big Five personalities to rule out potential alternative explanations. We measured the other four dimensions of Big Five personality traits using Mini-Markers (Saucier, 1994; 1 = *extremely inaccurate* to 7 = *extremely accurate*). Each personality trait measure contained eight items. Participants rated how extensively each item described their personality traits. The Cronbach’s alpha coefficients for openness, conscientiousness, agreeableness, and neuroticism were 0.82, 0.80, 0.77, and 0.81, respectively. We also controlled for possible effects of age and gender known to be related to socialization outcomes (e.g., Allen and Meyer, 1990; Scandura and Lankau, 1997). Of note, our hypotheses tests demonstrated the same pattern of findings regardless of whether we included the control variables of openness, conscientiousness, agreeableness, and neuroticism.

## Results

### Descriptive Statistics and Confirmatory Factor Analysis

Means, SDs, and inter-correlations among all study variables are presented in Table 1. Before testing hypotheses, we followed previous studies (Li et al., 2018; Lin et al., 2020, 2021) and conducted model comparisons using a series of confirmatory factor analyses to examine the distinctiveness of our focal variables. All analyses were conducted with Mplus 8.3 (Muthén and Muthén, 2017). Three parcels of items were constructed for each factor using a random assignment procedure. The hypothesized seven-factor (i.e., extraversion, social acceptance, depression, openness, conscientiousness, agreeableness, and neuroticism) measurement model provided a good fit to the data [*χ*^2^/*df* (483.16/168) = 2.88; CFI = 0.92; TFI = 0.90; root mean square error of approximation (RMSEA = 0.07)]. All factor loadings for items were significant (*p* < 0.001). Then, we compared the hypothesized seven-factor model (i.e., extraversion, social acceptance, depression, openness, conscientiousness, agreeableness, and neuroticism) with 21 alternative six-factor models in which any two of the seven factors were combined. Results showed that the hypothesized seven-factor model fit the data significantly better than any of the 21 six-factor models [Δ*χ*^2^ (Δ*df* = 6) ranged from 56.76 to 589.43, *p* < 0.01], suggesting that any two of the seven factors cannot be combined. These results offer support for the discriminant validity of our focal variables (see more details in Table 2).

**Table 1:** Descriptive statistics, reliabilities, and intercorrelations among study variables.

	Mean	SD	1	2	3	4	5	6	7	8	9
1 Gender	–	–									
2 Age	18.10	0.69	−0.03								
3 Openness	5.10	0.83	0.08	−0.03	(0.82)						
4 Conscientiousness	4.88	0.85	0.08	0.01	0.44^**^	(0.80)					
5 Agreeableness	2.48	0.74	−0.02	0.01	0.41^**^	0.38^**^	(0.77)				
6 Neuroticism	3.14	1.02	−0.06	−0.02	−0.37^**^	−0.41^**^	−0.57^**^	(0.81)			
7 Extraversion	4.27	1.04	−0.10	0.03	0.42^**^	0.16^**^	0.43^**^	−0.37^**^	(0.84)		
8 Social acceptance	4.64	0.86	−0.05	−0.01	0.27^**^	0.29^**^	0.43^**^	−0.41^**^	0.43^**^	(0.86)	
9 Depression	1.89	0.51	−0.06	−0.03	−0.29^**^	−0.20^**^	−0.32^**^	0.40^**^	−0.32^**^	−0.50^**^	(0.86)

*N* = 371; alpha reliabilities are provided in parentheses on the diagonal.

** *p* < 0.01 (two-tailed).

**Table 2:** Fit indices of measurement models.

Measurement models	*χ*^2^	*df*	CFI	TFI	RMSEA	Δ*χ*^2^ (*Δdf*)
Hypothesized seven-factor model	483.16	168	0.92	0.90	0.07	
**Alternative six-factor models (combining any two of the seven factors)**						
Model 1 (combining extraversion and social acceptance)	762.57	174	0.85	0.82	0.10	279.41^**^ (6)^a^
Model 2 (combining extraversion and depression)	800.05	174	0.84	0.81	0.10	316.89^**^ (6)^a^
Model 3 (combining openness and social acceptance)	965.00	174	0.80	0.76	0.11	481.84^**^ (6)^a^
Model 4 (combining openness and depression)	974.17	174	0.80	0.76	0.11	491.01^**^ (6)^a^
Model 5 (combining conscientiousness and social acceptance)	1034.20	174	0.79	0.74	0.12	551.04^**^ (6)^a^
Model 6 (combining conscientiousness and depression)	1072.59	174	0.78	0.73	0.12	589.43^**^ (6)^a^
Model 7 (combining agreeableness and social acceptance)	680.21	174	0.87	0.85	0.09	197.05^**^ (6)^a^
Model 8 (combining agreeableness and depression)	752.04	174	0.86	0.83	0.10	268.88^**^ (6)^a^
Model 9 (combining neuroticism and social acceptance)	836.45	174	0.84	0.80	0.10	353.29^**^ (6)^a^
Model 10 (combining neuroticism and depression)	885.79	174	0.82	0.79	0.11	402.63^**^ (6)^a^
Model 11 (combining extraversion and openness)	719.43	174	0.86	0.84	0.09	236.27^**^ (6)^a^
Model 12 (combining extraversion and conscientiousness)	844.48	174	0.83	0.80	0.10	361.32^**^ (6)^a^
Model 13 (combining extraversion and agreeableness)	688.51	174	0.87	0.85	0.09	205.35^**^ (6)^a^
Model 14 (combining extraversion and neuroticism)	751.97	174	0.86	0.83	0.10	268.81^**^ (6)^a^
Model 15 (combining openness and conscientiousness)	838.04	174	0.83	0.80	0.10	354.88^**^ (6)^a^
Model 16 (combining openness and agreeableness)	676.02	174	0.88	0.85	0.09	192.86^**^ (6)^a^
Model 17 (combining openness and neuroticism)	797.77	174	0.84	0.81	0.10	314.61^**^ (6)^a^
Model 18 (combining conscientiousness and agreeableness)	761.68	174	0.85	0.82	0.10	278.52^**^ (6)^a^
Model 19 (combining conscientiousness and neuroticism)	907.94	174	0.82	0.78	0.11	424.78^**^ (6)^a^
Model 20 (combining agreeableness and neuroticism)	539.92	174	0.91	0.89	0.08	56.76^**^ (6)^a^
Model 21 (combining social acceptance and depression)	886.40	174	0.82	0.79	0.11	403.24^**^ (6)^a^

*N* = 371; *χ*^2^ = chi-square; RMSEA, root mean square error of approximation; CFI, comparative fit index; TLI, Tucker–Lewis index.

** *p* < 0.01.

a Indicates model comparison to the seven-factor model.

### Hypothesis Testing

To test extraversion’s curvilinear effect on social acceptance and depression, we first estimated a linear model in which we regressed social acceptance and depression on Big Five personality traits, age, and gender. The model accounted for 32% of the variance in social acceptance and 23% of the variance in depression. Table 3 shows unstandardized coefficient estimates for Model 1. Based on Model 1, we added the effects of the extraversion-squared term on social acceptance and depression (Model 2) to test Hypothesis 1. In order to reduce multicollinearity concerns, we mean-centered extraversion before computing the squared term of extraversion (Aiken and West, 1991).

**Table 3:** Unstandardized regression coefficients.

	Model 1		Model 2	
	Social acceptance	Depression	Social acceptance	Depression
Constant	5.76^**^ (1.06)	1.85^**^ (0.66)	5.73^**^ (1.05)	1.87^**^ (0.65)
Gender	−0.07 (0.08)	−0.05 (0.05)	−0.08 (0.08)	−0.05 (0.05)
Age	−0.04 (0.05)	−0.02 (0.03)	−0.03 (0.05)	−0.02 (0.03)
Openness	−0.02 (0.06)	−0.05 (0.03)	−0.02 (0.05)	−0.05 (0.03)
Conscientiousness	0.14^**^ (0.05)	0.01 (0.03)	0.14^**^ (0.05)	0.01 (0.03)
Agreeableness	0.19^**^ (0.07)	−0.04 (0.04)	0.19^**^ (0.06)	−0.04 (0.04)
Neuroticism	−0.16^**^ (0.05)	0.14^**^ (0.03)	−0.16^**^ (0.05)	0.14^**^ (0.03)
Extraversion	0.22^**^ (0.04)	−0.08^**^ (0.03)	0.22^**^ (0.04)	−0.08^**^ (0.03)
Extraversion-squared			−0.07^*^ (0.03)	0.04^*^ (0.02)
*R*^2^	0.315^**^	0.231^**^	0.327^**^	0.240^**^
Δ*R*^2^			0.012^*^	0.009^*^
*F*	23.426^**^	15.340^**^	6.114^*^	4.099^*^

*N* = 371. Unstandardized coefficients are presented. SEs are reported in parentheses.

* *p* < 0.05;

** *p* < 0.01 (two-tailed).

**Figure 1:**
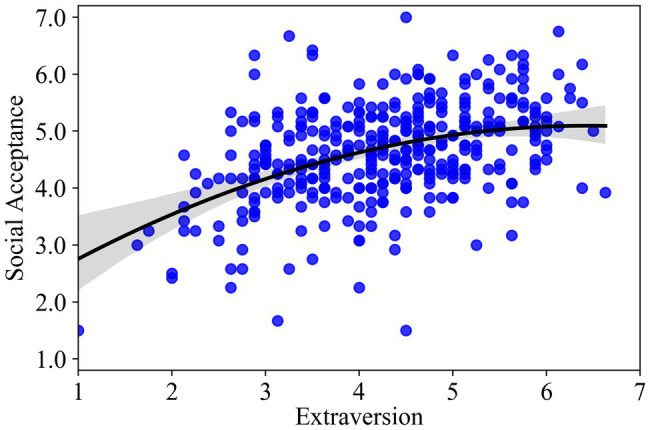
Curvilinear relationship between extraversion and social acceptance.

As seen in Table 3, extraversion was positively related to social acceptance (estimate = 0.22, standard error (SE) = 0.04, *p* < 0.01), but the coefficient for extraversion-squared was negatively related to social acceptance (estimate = −0.07, SE = 0.03, *p* < 0.05), indicating an inverted U-shaped curve with an overall positive trend (Aiken and West, 1991). Model 2 accounted for 33% of the variance in social acceptance, with the squared term of extraversion explaining additional 1.2% of the variance beyond Model 1 (*F* = 6.11, *p* < 0.05). After excluding the control variables of openness, conscientiousness, agreeableness, and neuroticism from the model, the coefficient for extraversion-squared was also negatively related to social acceptance (estimate = −0.08, SE = 0.03, *p* < 0.01), the squared term of extraversion explained additional 1.6% of the variance beyond Model 1. The curve is presented in Figure 1, which also includes the individual data points. Simple slope analyses demonstrated that the effect of extraversion on social acceptance was significantly positive at the low level (−1 SD; *B* = 0.36, SE = 0.07, *p* < 0.001) and at the average level (*B* = 0.22, SE = 0.04, *p* < 0.001) of extraversion, but not significant at the high level (+1 SD) of extraversion (*B* = 0.07, SE = 0.07, *p* = 0.32).

**Figure 2:**
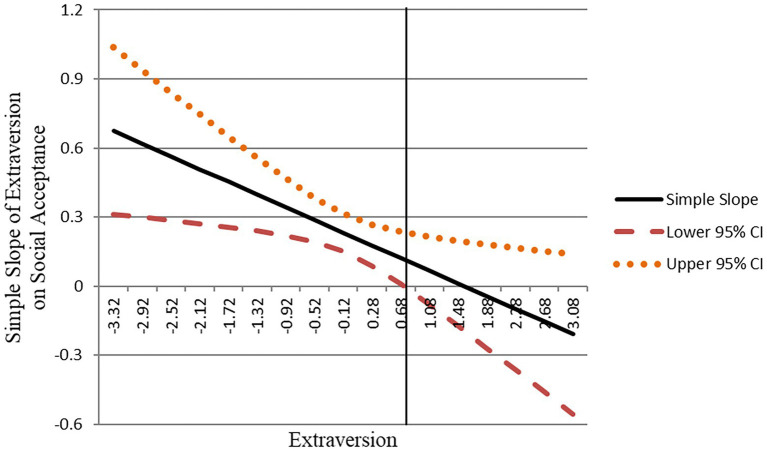
Johnson–Neyman (J–N) plot of the region of significance for the simple slope of extraversion on social acceptance.

Then, we used the Johnson–Neyman (J–N) technique (Miller et al., 2013) to analyze the region of significance for the curvilinear effect. Based on the J–N plot (Figure 2), it is shown that when extraversion was lower (specifically less than 0.718 units), the relationship between extraversion and social acceptance was significantly positive, indicating that when extraversion was below 0.718 units, an increase in extraversion would result in a statistically significant increase in social acceptance. When extraversion is above 0.718 units, the relationship between extraversion and social acceptance was not significant, suggesting that when extraversion exceeds 0.718 units, an increase in extraversion would not lead to a significant increase in social acceptance. Thus, Hypothesis 1 was supported.

**Figure 3:**
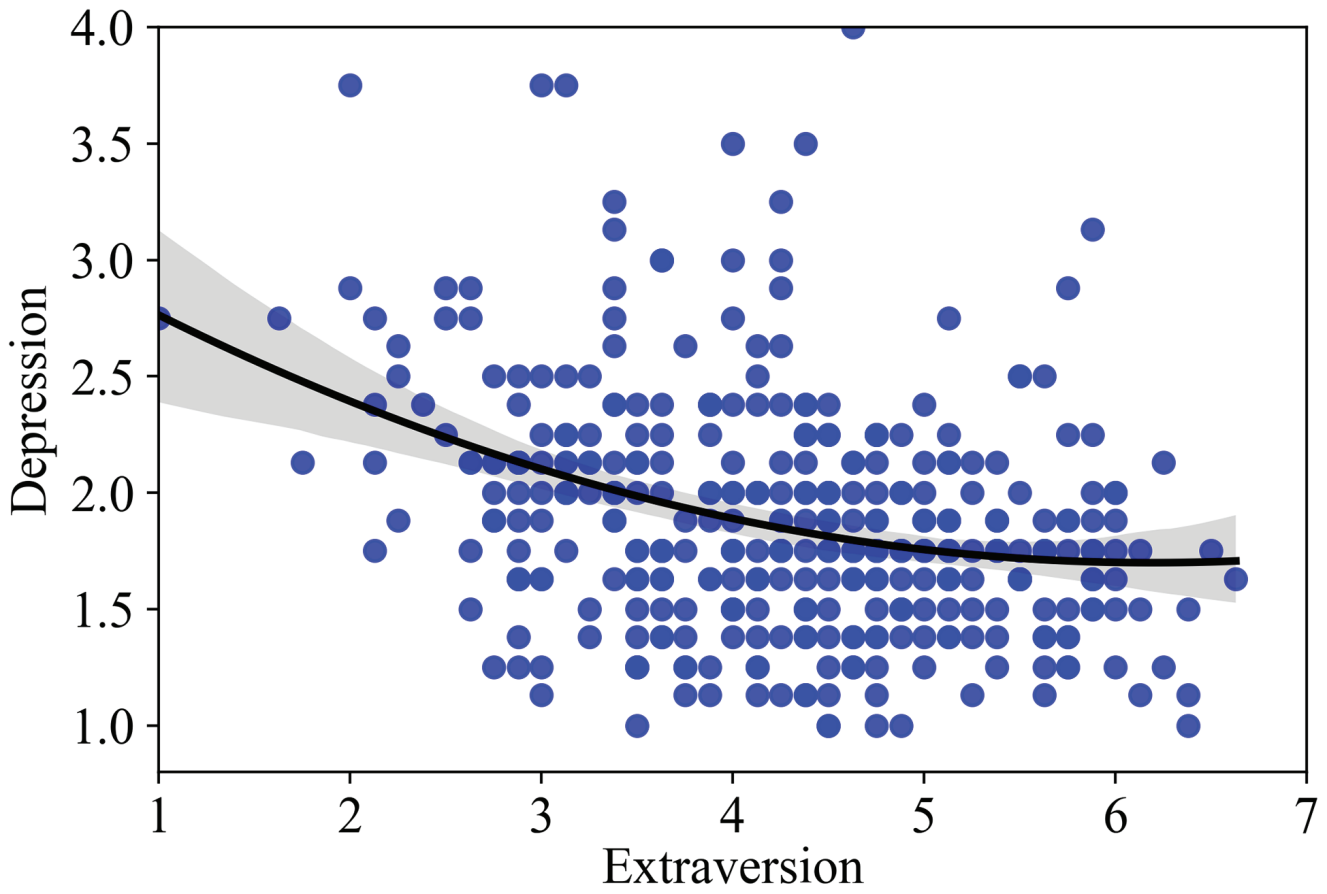
Curvilinear relationship between extraversion and depression.

As seen in Table 3, extraversion was negatively related to depression (estimate = −0.08, SE = 0.03, *p* < 0.01). The coefficient for extraversion-squared was positively related to depression (estimate = 0.04, SE = 0.02, *p* < 0.05), indicating a U-shaped curve with an overall negative trend (Aiken and West, 1991). Model 2 accounted for 24% of the variance in depression, with the squared term of extraversion explaining additional 0.9% of the variance beyond Model 1 (*F* = 4.01, *p* < 0.05). After excluding the control variables of openness, conscientiousness, agreeableness, and neuroticism from the model, the coefficient for extraversion-squared was also positively related to depression (estimate = 0.04, SE = 0.02, *p* < 0.05), and the squared term of extraversion explained additional 1.3% of the variance beyond Model 1. The curve is presented in Figure 3, which also includes the individual data points. Simple slope analyses demonstrated that the effect of extraversion on depression was significantly negative at the low level (−1 SD; *B* = −0.15, SE = 0.04, *p* < 0.001) and at the average level (*B* = −0.08, SE = 0.03, *p* < 0.01) of extraversion, but not significant at the high level (+1 SD; *B* = −0.00, SE = 0.05, *p* = 0.93) of extraversion.

**Figure 4:**
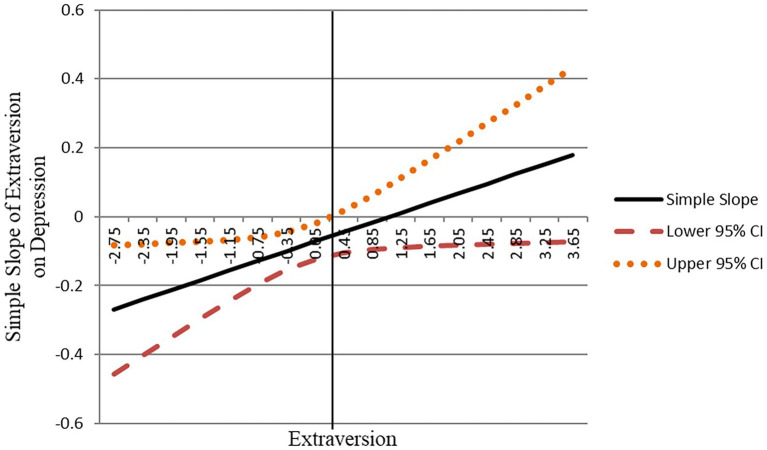
Johnson–Neyman plot of the region of significance for the simple slope of extraversion on depression.

Later, we used the J–N technique (Miller et al., 2013) to analyze the region of significance for the curvilinear effect. Based on the J–N plot (Figure 4), it is shown that when extraversion was lower (specifically less than 0.292 units), the simple slope for extraversion predicting depression was significantly negative, indicating that when extraversion was below 0.292 units, an increase in extraversion would result in a statistically significant decrease in depression. When extraversion is above 0.292 units, however, an increase in extraversion would not lead to any significant change in depression. Thus, Hypothesis 2 was supported.

## Discussion

Based on the TMGT effect, our findings showed that extraversion is curvilinearly related to the social acceptance and depression, which offers a better understanding of the mechanism through which personality trait influences the socialization outcomes. Specifically, extraversion had a curvilinear relationship with social acceptance, such that the relationship was significantly positive from lower to moderate levels of extraversion, but the positive relationship leveled off at higher levels of extraversion. Moreover, extraversion also had a curvilinear relationship with depression, such that the relationship was significantly negative from lower to moderate levels of extraversion, but the negative relationship leveled off at higher levels of extraversion. We demonstrate that beyond a certain point, the beneficial effects of extraversion on socialization outcomes were diminished. That is, higher levels of extraversion were not associated with more positive socialization outcomes (though they were not associated with worse outcomes either) when extraversion exceeded a certain point.

### Theoretical Implications

Although the variances explained uniquely by the squared term of extraversion indicates a relatively small effect size for the curvilinear effects, personality scholars highlighted that “any increase in the predictive validity of personality measures is a benefit, especially when there are no additional costs associated with the increased validity” (Le et al., 2011, p. 127). Thus, the potential implications of such incremental validity in predicting subsequent outcomes should be given attention (Funder and Ozer, 2019).

First, we contribute to the personality theory that challenges “theoretical consensus” in extraversion literature and demonstrates that more is not necessarily better. Extraverted students were once expected to have positive socialization outcomes in terms of better social acceptance and lower level of depression (e.g., Ashton et al., 2002; Lubbers et al., 2006; Lee et al., 2008). However, extraversion has been shown to have cost-benefit tradeoffs (Lukaszewski and von Rueden, 2015; Jacques-Hamilton et al., 2018). Highly extraverted students might be excessively assertive, dominant, and eager to be at the center of social attention (Depue and Collins, 1999; Ashton et al., 2002; Roberts et al., 2006; Shao et al., 2013; Hu et al., 2019), which may not be helpful in increasing social acceptance and reducing depression. Our findings offer some new insights by showing that beyond a certain point, higher levels of extraversion are not associated with more positive socialization outcomes, though they are not associated with worse outcomes either.

Moreover, an important implication of the TMGT effect of personality on desired socialization outcomes could put forward our exploration of the threshold of context-specific inflection points. As such, we encourage future research to examine the buffering role of effective moderators on the curvilinear relationships between extraversion and socialization outcomes. For instance, when individuals have high levels of emotional competence (Szczygiel and Mikolajczak, 2018) or prosocial motivation (Hu et al., 2019), extraversion may have a prolonged and strengthened positive effect on social interaction.

Third, we also enhance more nuanced understandings of the socialization process. University freshmen face “reality shocks” when they confront new academic, social, and emotional challenges (Chickering, 1969; Mattanah et al., 2010; Klimstra et al., 2018; Deng and Yao, 2020). However, existing curvilinear relationship findings mainly focused on the relationship between personality factors and task-relevant performance (Le et al., 2011; Carter et al., 2014), few researchers concern about the curvilinear effects of personality factors on socialization outcomes beyond task. Given that extraversion has a strong relation to interpersonal relationship and psychological wellbeing (Costa and McCrae, 1980; Hogan et al., 1997; Jensen-Campbell et al., 2002; Lubbers et al., 2006; Lee et al., 2008), our study supplements the research of the relationship between important personality factor in the Big Five personality traits framework and a broader set of socialization outcomes by examining the curvilinear effects of extraversion on social acceptance and depression among freshmen.

### Practical Implications

Our findings have several practical implications for universities and organizations. First, knowledge about the curvilinear relationship between personality and socialization outcomes could be used to improve personnel selection practices. For instance, selection based on cutoff points should be more appropriate: after a certain point, personality would not always be positively (or negatively) correlated to some socialization performance. As such, student admissions officers or/and human resource managers are advised to adopt rational views about extraversion, particularly those who highly value extraversion in students or applicants. It seems more appropriate that personality tests, based on a double cutoff strategy with both lower and upper limit settings (Le et al., 2011), should be used earlier while selecting applicants. Second, our findings may contribute to alleviating the risk of faking in the personnel selection. Applicants tend to exaggerate their personality aptitude scores during the assessments because of the social desirability (Podsakoff et al., 2003). Using a double cutoff strategy with both lower and upper limit settings could help exclude applicants with extremely high scores, regardless of whether the scores are reliable or faked (Le et al., 2011). Of note, we offer the above-mentioned practical implications with caution that even though the curvilinear effects we examined were all statistically significant and support our hypotheses, some of them had a small variance for the changed *R*^2^ (see Table 3), likely indicating small effect sizes. As such, we do not attempt to discount the importance of extraversion in the socialization process; rather, we take a more nuanced view by drawing the educational and organizational attention of managers to the effects of higher levels of extraversion on social interaction and psychological wellbeing of individuals. Thus, the current findings should be applied with some caution.

### Limitations and Future Research

Future research should address several limitations of this study. First, our sample may have limited generalizability because it only comprised students in a university. Furthermore, although university and organizational socialization processes are similar (Wang et al., 2013; Deng and Yao, 2020), whether our findings could generalize to the workplace and other contexts needs to be examined. Moreover, as freshmen spend most of their time with schoolmates in the university (e.g., they may share common courses, communities, or even residence with other students), general socializing with schoolmates is a basis for the freshmen to develop friendship network, share resources and information, and receive support, which can facilitate adaptation of freshmen to the university (Wang et al., 2013; Deng and Yao, 2020). As such, it is possible that freshmen behave in a more extraverted fashion than their actual level of extraversion during the transition period. Thus, future research could measure behavioral manifestations of extraversion, such as the frequency of general socializing behavior (Ashford and Black, 1996) to more accurately capture the characteristics of extraversion and its influences on newcomer socialization. Second, collecting data exclusively in the Chinese cultural context is another limitation. China is highly collectivistic (Hofstede, 2001) and strongly emphasizes conformity and interpersonal reliance (Wang et al., 2013). Being overly assertive and outgoing may not necessarily result in receiving positive peer reactions. In contrast, students in an individualistic culture are expected and encouraged to communicate in a more assertive way in public (Tavakoli et al., 2009). Thus, Chinese students who are highly extraverted may lose advantages on having better social interaction and experiencing more positive emotions, whereas students in Western contexts may not have such interpersonal risks. Future research should revalidate our findings in more diversified contexts. Third, we used self-reported measures of personality traits and socialization outcomes, so our findings may suffer from common method bias (Podsakoff et al., 2003), though concerns are somewhat reduced by the interval of the data collection across 3 months. Fourth, we focused only on extraversion and ignored other likely predictors. Conscientiousness and neuroticism have been found to be curvilinearly related to some behavioral outcomes (Le et al., 2011; Carter et al., 2014; Uppal, 2017; Yuan et al., 2018). Future research could consider other personality factors such as conscientiousness, neuroticism, agreeableness, and openness as possible predictors of social acceptance and psychological wellbeing. In addition, given that the Mini-marker measure of extraversion has found to be reliable and valid (e.g., Diefendorff, 2003; Bauer et al., 2006; Kiffin-Petersen et al., 2011; Li and Xu, 2020; Spark and O’Connor, 2020), we used this brief measure to reduce burden of participants in completing the surveys. Future research could revalidate our findings using other full scales (e.g., NEO-PI-R, Costa and McCrae, 1992) to measure Big Five personality traits.

## Data Availability Statement

The raw data supporting the conclusions of this article will be made available by the authors, without undue reservation.

## Ethics Statement

The studies involving human participants were reviewed and approved by Department of Psychology. The patients/participants provided their written informed consent to participate in this study.

## Author Contributions

YD: methodology, software, data curation, validation, formal analysis, investigation, writing-original draft, writing-reviewing and editing, and visualizing. HC: writing-original draft, writing-reviewing and editing, and visualizing. XY: conceptualization, resources, data curation, supervision, writing-reviewing and editing, project administration, and funding acquisition. All authors contributed to the article and approved the submitted version.

### Conflict of Interest

The authors declare that the research was conducted in the absence of any commercial or financial relationships that could be construed as a potential conflict of interest.
